# Accurate Coil Positioning is Important for Single and Paired Pulse TMS on the Subject Level

**DOI:** 10.1007/s10548-018-0655-6

**Published:** 2018-06-25

**Authors:** Annika A. de Goede, Esther M. ter Braack, Michel J. A. M. van Putten

**Affiliations:** 1 Department of Clinical Neurophysiology, Technical Medical Centre, University of Twente, Carré 3.714, P.O. Box 217, 7500 AE Enschede, The Netherlands; 2 Department of Neurology and Clinical Neurophysiology, Medisch Spectrum Twente, Enschede, The Netherlands

**Keywords:** Transcranial magnetic stimulation, TMS, Coil positioning, Location, Orientation, Function-guided navigation

## Abstract

Function-guided navigation is commonly used when assessing cortical excitability using transcranial magnetic stimulation (TMS). However, the required accuracy, stability and the effect of a change in coil positioning are not entirely known. This study investigates the accuracy of function-guided navigation for determining the hotspot. Furthermore, it evaluates the effect of a change in coil location on the single and paired pulse excitability measures: motor evoked potential (MEP) amplitude, TMS evoked potential (TEP) and long intracortical inhibition (LICI), and of a change in coil orientation on LICI. Eight healthy subjects participated in the single pulse study, and ten in the paired pulse study. A robot-guided navigation system was used to ensure accurate and stable coil positioning at the motor hotspot as determined using function-guided navigation. In addition, we targeted four locations at 2 mm and four at 5 mm distance around the initially defined hotspot, and we increased and decreased the coil orientation by 10°. In none of the subjects, the largest MEP amplitudes were evoked at the originally determined hotspot, resulting in a poor accuracy of function-guided navigation. At the group level, a change in coil location had no significant effect on the MEP amplitude, TEP, or LICI, and a change in coil orientation did not significantly affected LICI. However, at the subject level significant effects on MEP amplitude, TEP, and LICI were found for changes in coil location or orientation, although absolute differences were relatively small and did not show a consistent pattern. This study indicates that a high accuracy in coil positioning is especially required to measure cortical excitability reliably in individual subjects using single or paired pulse TMS.

**Electronic supplementary material:**

The online version of this article (10.1007/s10548-018-0655-6) contains supplementary material, which is available to authorized users.

## Introduction

Transcranial magnetic stimulation (TMS) is a non-invasive technique for assessing cortical excitability (Barker et al. 1985). Initially, only the integrated corticospinal excitability could be measured by combining single pulse TMS with electromyography (EMG) (Valls-Solé et al. 1992; Abbruzzese and Trompetto 2002). However, paired pulse TMS-EMG focuses more on the excitability of cortical neurons (Kujirai et al. 1993; Abbruzzese and Trompetto 2002), while TMS combined with electroencephalography (EEG) measures the direct neuronal response (Ilmoniemi et al. 1997; Ilmoniemi and Kičić 2010). Although TMS is used to study a variety of neuropsychiatric conditions (Chen et al. 2008; Ni and Chen 2015), it is only routinely used for therapeutic purposes. The applicability of TMS as a clinical tool for diagnostics or therapy evaluation is limited, mainly due to a high intra-subject and inter-subject variability of excitability measures (Wassermann 2002; Ni and Chen 2015).

Part of the intra-subject and inter-subject variability is caused by fluctuations in physiological processes (Schmidt et al. 2015; Goldsworthy et al. 2016). However, it is difficult to control these processes, such as the level of muscle pre-activation (Hess et al. 1987; Darling et al. 2006), the state of ongoing cortical oscillatory rhythms (Sauseng et al. 2009; Bergmann et al. 2012), and both the attention level and arousal state of participants (Mars et al. 2007). Non-biological causes of variation are easier to address. For example, the variability of excitability measures can be reduced by minimizing external noise, increasing the number of trials (Goldsworthy et al. 2016; Chang et al. 2016), and optimizing the coil positioning, in terms of location, orientation and tilt (Hess et al. 1987; Amassian et al. 1989; Schmidt et al. 2015). Of these three suggestions, accurate and stable positioning of the coil is probably the most difficult to achieve, while its contribution to reducing variability is largely unknown (Schmidt et al. 2015).

Several navigation methods can be used to determine the coil location, while the coil is placed by default at 45° from the midline (orientation) and tangentially to the stimulation target (tilt) (Groppa et al. 2012). The traditional function-guided method uses signature outputs, such as motor responses, to locate a hotspot in the primary motor cortex (Barker et al. 1985; Rossini et al. 2015). To determine the hotspot for a particular target muscle, the coil is moved gradually over the motor cortex to find the location that evokes the largest EMG responses, while applying a series of pulses at a relatively high intensity (Rossini et al. 1994, 2015). The hotspot is not only used as stimulation location in TMS-EMG studies, but is also a preferred target for TMS-EEG. When other targets are to be stimulated, such as the dorsolateral prefrontal cortex, the hotspot is first targeted for evaluating the motor threshold and determining the stimulation intensity (Komssi et al. 2004, 2007; Kähkönen et al. 2005). Therefore, correct positioning of the coil at the hotspot is important for a broad range of TMS studies.

Alternatively, neuronavigation methods make use of individual brain imaging data to position the coil above a selected cortical area (Schönfeldt-Lecuona et al. 2005; Sparing et al. 2008; Lefaucheur 2010). It is often combined with a frameless stereotaxic system to not only ensure accurate positioning of the coil, but also coil stability throughout the TMS session (Sparing et al. 2010; Lefaucheur 2010; Cincotta et al. 2010). Despite the high accuracy and stability of neuronavigation (Herwig et al. 2001; Sparing et al. 2010; Lefaucheur 2010), function-guided navigation is still a commonly used method to determine the hotspot since it can be easily performed. However, little is known about the accuracy and stability required for coil positioning during both single and paired pulse TMS, and about the effect of a change in coil positioning, in terms of location, orientation and tilt.

In this study, we investigate the accuracy of function-guided navigation for determining the hotspot. We evaluate the effect of a 2 or 5 mm change in coil location on the MEP amplitude, TMS evoked potential (TEP) and long intracortical inhibition (LICI). In addition, we evaluate the effect of a 10° change in coil orientation on LICI. Furthermore, we investigate the stability of these single and paired pulse TMS parameters at different locations at and around the hotspot. The hotspot was determined using function-guided navigation, after which a robot-guided navigation system was used to ensure accurate and stable coil positioning during stimulation.

## Materials and Methods

Single and paired pulse TMS data was collected as part of two larger trials (Trial ID: NL36317.044.11 for single pulse data and Trial ID: NL49854.044.14 for paired pulse data). Both study protocols were approved by the local medical ethics committee (Medisch Spectrum Twente, Enschede, The Netherlands) and were in accordance with the Declaration of Helsinki. We followed the guidelines for the use of TMS in clinical practice and research (Rossi et al. 2009). Part of the dataset was previously used in another context by ter Braack et al. (2013b) and by de Goede and van Putten (2017).

### Subjects

Healthy adults (aged > 18 years) were included after giving written informed consent and filling out the Screening Questionnaire before TMS (Rossi et al. 2011) and the Dutch Handedness Questionnaire (van Strien 1992, 2003). Subjects with contraindications to TMS were excluded. Eight subjects (seven males, mean age 24 ± 1.6 years; range 23–27 years, all right-handed) were included in the single pulse TMS study, and another ten subjects (four males, mean age 28 ± 8.8 years; range 22–51 years, nine right-handed) in the paired pulse TMS study. In the single pulse TMS study, EMG and EEG data was obtained simultaneously, while in the paired pulse TMS study only EMG data was measured.

### Coil Positioning

Positioning of the TMS coil, with an optical tracking accuracy of 1 mm in every direction, was achieved using a robot-guided navigation system (ANT Neuro, Enschede, The Netherlands (ANT-neuro.com)). The position of the robot, coil and subject were continuously tracked by a Polaris infrared camera system (Northern Digital, Waterloo, Canada). Through a calibration procedure the robot, TMS coil and tracking system were registered to a common coordinate system. Subjects were tracked using a headband with four passive reflective markers. A standard 1.5 T magnetic resonance image was used to create a head model which was linked to the subject by collecting three landmarks and approximately 300 additional points on the scalp with a tracking pointer. We used a robotic arm holding the coil for accurate positioning at the stimulation target. Displacements from the target were automatically detected and actively corrected by the robotic arm to ensure accurate and stable coil positioning throughout the TMS session.

### Stimulation Target: Motor Hotspot

**Fig. 1:**
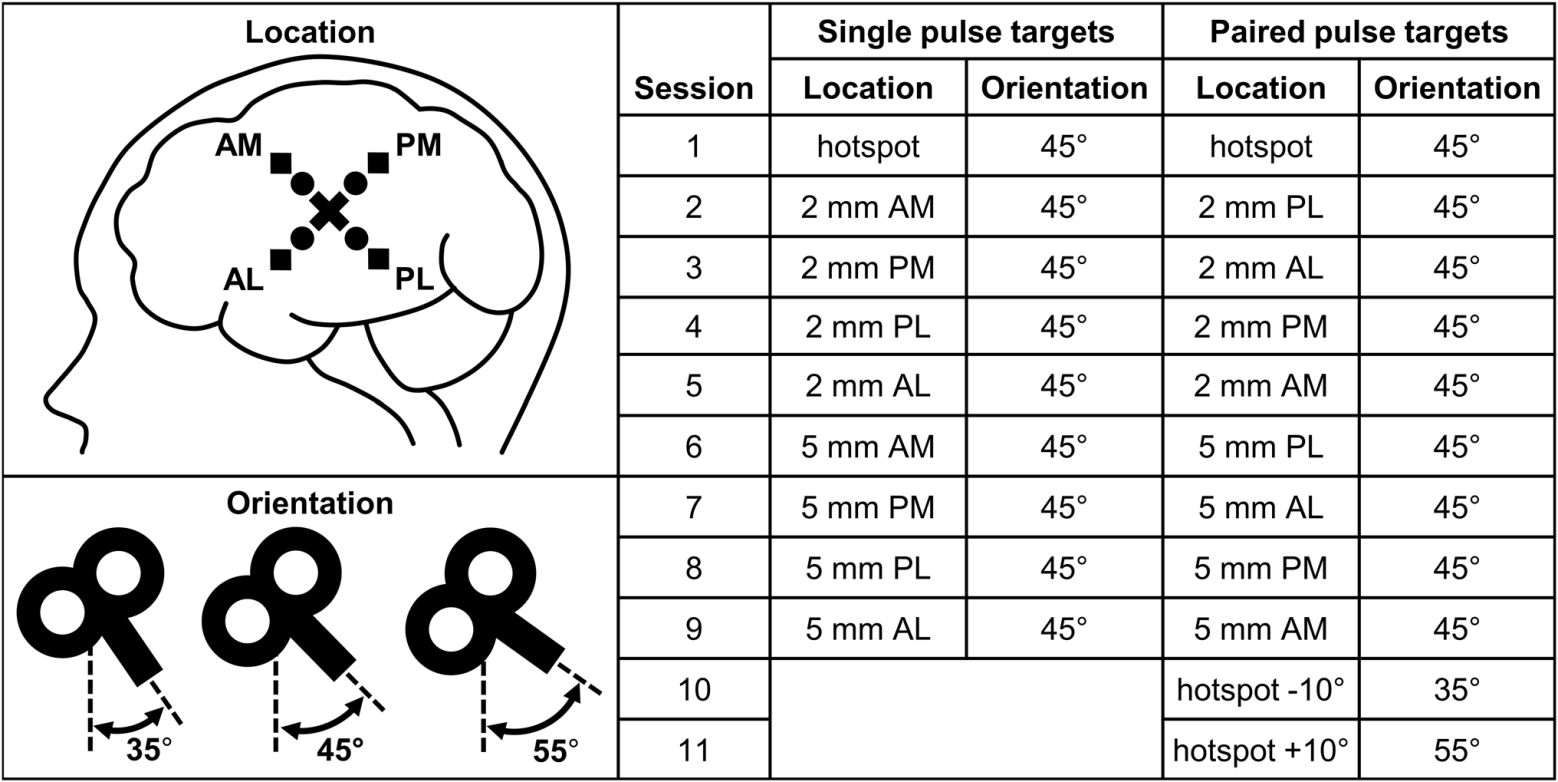
Overview of the stimulated single and paired pulse targets. The stimulation locations are represented by a cross for the hotspot, by circles for the four locations at a distance of 2 mm from the hotspot, and by squares for the four locations at a distance of 5 mm from the hotspot. *AM* anterior-medial, *PM* posterior-medial, *PL* posterior-lateral, and *AL* anterior-lateral

In all subjects, the left motor hotspot of the abductor digiti minimi (ADM) muscle was the primary stimulation target. The hotspot was located by manual function-guided navigation. The location in the motor cortex that evoked the largest MEPs was marked on the created head model, which was linked to the subject. Hereafter robot-guided navigation was used for stable coil positioning at the indicated hotspot. The TMS coil was placed tangentially at the ADM hotspot, with the handle pointing backwards and laterally at an angle of 45° from the midline. In both the single and paired pulse TMS study, the hotspot was stimulated at the start of the study (session 1). For an overview of the stimulated targets, see Fig. 1.

### Change in Coil Location

In addition to the hotspot, we targeted four locations at a distance of 2 mm and four locations at a distance of 5 mm from the hotspot, see Fig. 1. The coil was either moved in an anterior-medial (AM), anterior-lateral (AL), posterior-medial (PM) or posterior-lateral (PL) direction. Except for this change in coil location, the orientation (45° from the midline) and tilt (tangentially to the stimulation target) were kept constant.

### Change in Coil Orientation

In the paired pulse study, we also evaluated the effect of a 10° change in coil orientation, see Fig. 1. The angle from the midline was decreased to 35° (session 10) and then increased to 55° (session 11). We only changed the coil orientation, while maintaining the same location (at the hotspot) and tilt (tangentially to the hotspot).

### Stimulation Protocol

Each subject was seated comfortably in a chair, with the right hand pronated in a relaxed position. Subjects were instructed to keep their eyes open and to look straight ahead during stimulation. In addition, we applied noise masking and placed a thin layer of foam between the coil and the head of the subject in the single pulse TMS study.

Biphasic TMS pulses, with pulse duration of 400 µs, were applied using a figure-of-eight air-cooled 70 mm coil and a Magstim Rapid^2^ Stimulator (both from The Magstim Company Ltd, Whitland, United Kingdom). The intensity of stimulation depended on the rMT, which was defined as the minimum intensity needed to evoke at least five MEPs of at least 50 µV out of ten consecutive pulses (Groppa et al. 2012; Rossini et al. 2015).

### Single Pulse TMS Protocol

Each target was stimulated by 75 single pulses at an intensity of 110% rMT, with a random inter-pulse interval between 3 and 4 s.

### Paired Pulse TMS Protocol

Each target was stimulated with ten paired pulses at five randomly applied interstimulus interval (ISIs): 100, 150, 200, 250 and 300 ms. Both the conditioning and test pulses were applied at an intensity of 120% rMT. A random interval between 3.5 and 4.5 s was kept between pairs of consecutive pulses.

### Electromyography Recording and Analysis

For the EMG recordings, two surface Ag/AgCl electrodes were placed in a belly-tendon montage over the right ADM muscle. The ground electrode was placed on the dorsal side of the hand. In the single pulse TMS study, we recorded the EMG using an additional amplifier coupled to a 64-channel EEG amplifier (both from TMSi, Oldenzaal, The Netherlands). In the paired pulse TMS study, the EMG was recorded using a 74-channel EEG amplifier (TMSi, Oldenzaal, The Netherlands). In both studies, EMG was sampled at 2048 Hz and low-pass filtered with an anti-aliasing filter with a cut-off frequency of 550 Hz.

Even though subjects were asked to fully relax their right ADM muscle, EMG recordings were afterwards checked for muscle pre-activation. Trials containing EMG activity larger than 50 µV in the 50 ms preceding a single or conditioning pulse were excluded in the single and paired pulse study, respectively.

### Single Pulse EMG Analysis: MEP Amplitude

For each subject and each target, we calculated the peak-to-peak amplitudes of the EMG responses. To perform statistical analysis at the group level, for each subject the mean MEP amplitude per target was taken.

### Paired Pulse EMG Analysis: LICI

LICI was determined separately for each subject, target and ISI. First, we calculated the mean peak-to-peak amplitude of the responses to the conditioning and test pulses. Next, we calculated the ratio between this mean amplitude of the test response (TR) and this mean amplitude of the conditioning response (CR), expressed as a percentage: 100 × TR/CR (%) (Valls-Solé et al. 1992). This ratio represents inhibition for values below 100% and facilitation for values above 100%.

### Electroencephalography Recording and Analysis

We continuously recorded the EEG during single pulse TMS using a 64-channel EEG amplifier (TMSi, Oldenzaal, The Netherlands) and a TMS-compatible EEG cap (ANT Neuro, Enschede, The Netherlands). The ground electrode was placed between electrode positions Fz and Fpz. The EEG was sampled at 2048 Hz and low-pass filtered with an anti-aliasing filter with a cut-off frequency of 550 Hz.

The EEG was analyzed in the common average montage. Trials were defined from 50 ms before to 350 ms after every TMS pulse, resulting in 75 trials of 400 ms for each electrode position. After removal of the baseline, single trail principal component analysis (PCA) was performed using 40 principal components. For a detailed description of the PCA methods see ter Braack et al. (2013a). The first four PCA components were removed, after which trials were filtered with a fourth order Butterworth bandpass filter between 1 and 45 Hz. Hereafter, trials were averaged, resulting in a TEP for each electrode position.

### Statistical Analysis

To evaluate the accuracy of function-guided navigation for determining the hotspot, we tested whether the highest MEP amplitudes were evoked at the hotspot. For the paired pulse study, the MEP amplitudes to the fifty conditioning pulses were taken. One-way ANOVA was used to test if there were significant differences in MEP amplitudes measured at the hotspot compared to the other stimulation targets, while Levene’s test was used to assess equality of variances.

To evaluate the effect of a change in coil location at the group level, one-way repeated measures ANOVA with Greenhouse-Geisser correction was used to test if there were significant differences in mean MEP amplitudes between the nine stimulation targets. To evaluate stability, we used Levene’s test to determine if there was a significant difference in mean MEP amplitude variance between targets. At the subject level, we used the outcomes of the one-way ANOVA and Levene’s test, as described in the first paragraph, to determine if there were significant differences in MEP amplitudes and amplitude variance between all nine targets.

To compare the EEG responses measured at the hotspot and at the locations around the hotspot, we used the nonparametric cluster-based permutation analysis (Maris and Oostenveld 2007) as implemented in the FieldTrip toolbox (http://www.fieldtriptoolbox.org). At the group level, dependent samples *t*-tests were used to compare the TEPs measured between two stimulation targets. Comparisons were performed for each electrode and each time sample between 0 and 300 ms. Only *t*-values with a *p*-value < 0.05 were clustered based on neighboring electrodes (n = 2) and adjacent time samples. The summed *t*-value of each cluster was statistically compared to the distribution of clusters obtained through a permutation test. Here, all trials were randomly assigned to either of the two locations for 1500 times (Monte Carlo estimate). Clusters in the original data were considered to be significant if less than 5% of the summed t-values obtained by permutation were larger than the cluster value observed in the original data. Analysis at the subject level was similar to the group level statistics, except that independent samples *t*-tests were used to compare the EEG trials measured at the nine stimulation locations at and around the hotspot.

To evaluate the effect of a change in coil location and orientation at the group level, one-way repeated measures ANOVA with Greenhouse-Geisser correction was used to test if there were significant differences in LICI measured at the eleven stimulation targets. Each ISI (100, 150, 200, 250 and 300 ms) was individually tested. To evaluate stability, we used Bartlett’s test to assess equality of variances. A non-parametric test was used as the data was not normally distributed. At the subject level, one-way ANOVA was used to test for significant differences between targets in LICI for each ISI, and Bartlett’s test for a significant difference in LICI variance. Propagation of uncertainty rules were applied to define the standard deviation (SD) belonging to a LICI ratio (Farrance and Frenkel 2012).

In case significant differences were found with the single or paired pulse TMS-EMG paradigms, we further evaluated the size of these differences to get insight into the impact of changes in coil location and orientation. Only for the significant target-comparisons we calculated the absolute difference in mean MEP amplitude or LICI between both targets and averaged these values.

For the statistical, as well as EMG and EEG analysis, we used Matlab (version R2015a, The Mathworks, Natick, MA, USA). For all statistical analysis *p*-values below 0.05 were considered statistically significant and additionally adjusted for the amount of performed tests (Bonferroni corrected with n = 36 and n = 55 for the single and paired pulse study, respectively). For the ANOVA’s, post hoc tests were performed to identify which targets differ from each other. Effect sizes were calculated using the partial eta-squared, defined as $\eta _{{\text{p}}}^{2}={\text{S}}{{\text{S}}_{{\text{effect}}}}/({\text{S}}{{\text{S}}_{{\text{effect}}}}\,+\,{\text{S}}{{\text{S}}_{{\text{error}}}}).$

## Results

In one subject (paired pulse nr. 10) the locations at 5 mm distance were skipped to shorten the measurement time because of discomfort, and in one subject (paired pulse nr. 8) coil orientation was not changed due to technical problems with the navigation system. All other participants tolerated the stimulation protocol well.

### Hotspot Accuracy

**Fig. 2:**
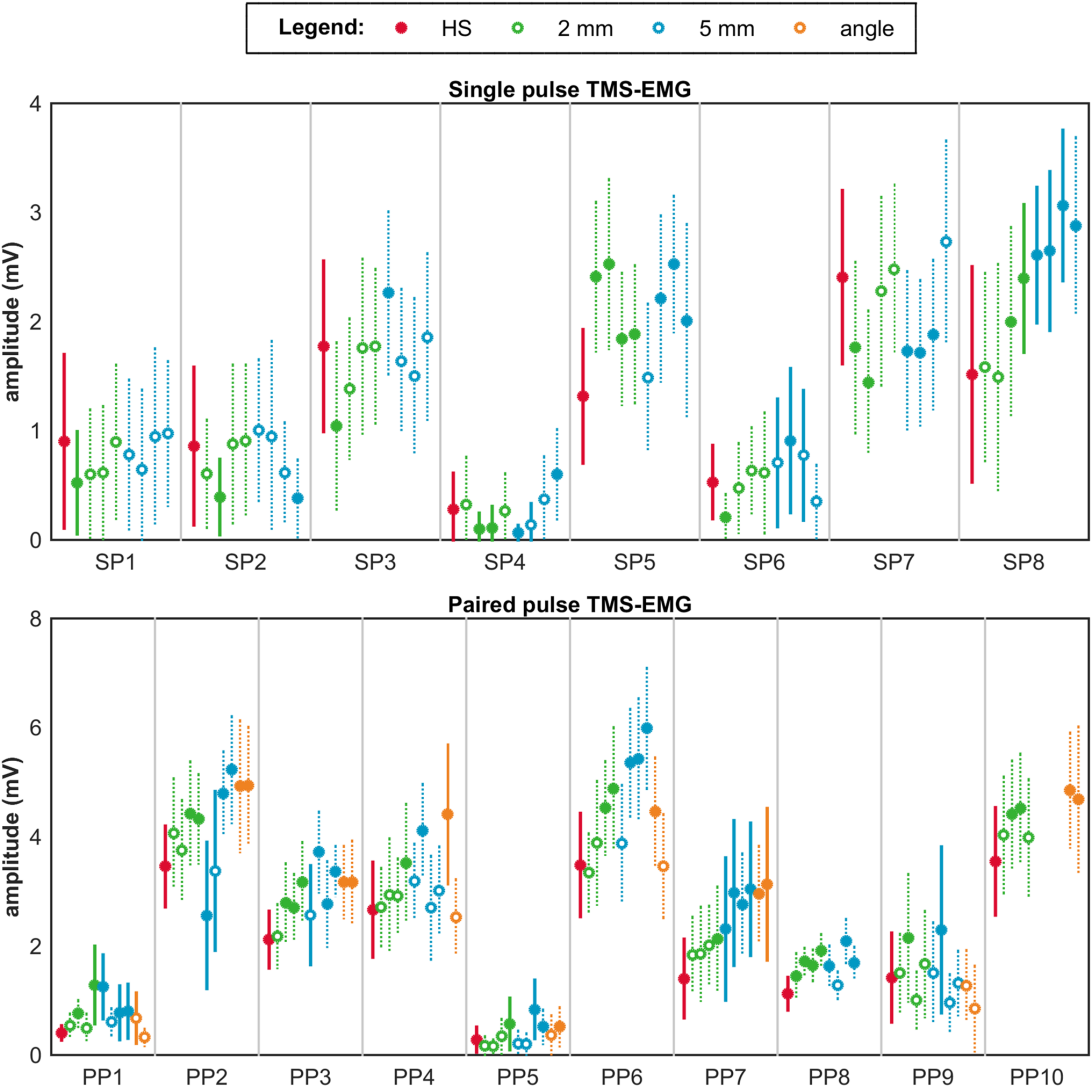
Outcomes of hotspot accuracy. The top row shows per subject the mean ± SD of the MEP amplitudes per target in the single pulse study. The bottom row shows per subject the mean ± SD of the conditioning MEP amplitudes per target in the paired pulse study. In red, the outcomes measured at the hotspot (HS); in green, at a distance of 2 mm from the hotspot (HS); in blue, at a distance of 5 mm from the hotspot (HS); and in orange, for a 10° change in coil orientation. Targets that differ significantly from the hotspot in mean MEP amplitude and amplitude variance are indicated with filled dots and straight lines, respectively

In all subjects, the largest MEP amplitudes were not evoked at the hotspot. In five out of eight subjects from the single pulse study and all ten subjects from the paired pulse study, amplitudes were significantly higher at another location compared to the hotspot, see filled dots in Fig. 2. In the single pulse study, five out of eight subjects showed inequality of variances, being significantly lower at the hotspot in only one. In seven out of ten subjects from the paired pulse study, a significantly lower variance was measured at the hotspot, see straight lines in Fig. 2.

### Single Pulse TMS-EMG Outcome: MEP Amplitude

**Fig. 3:**
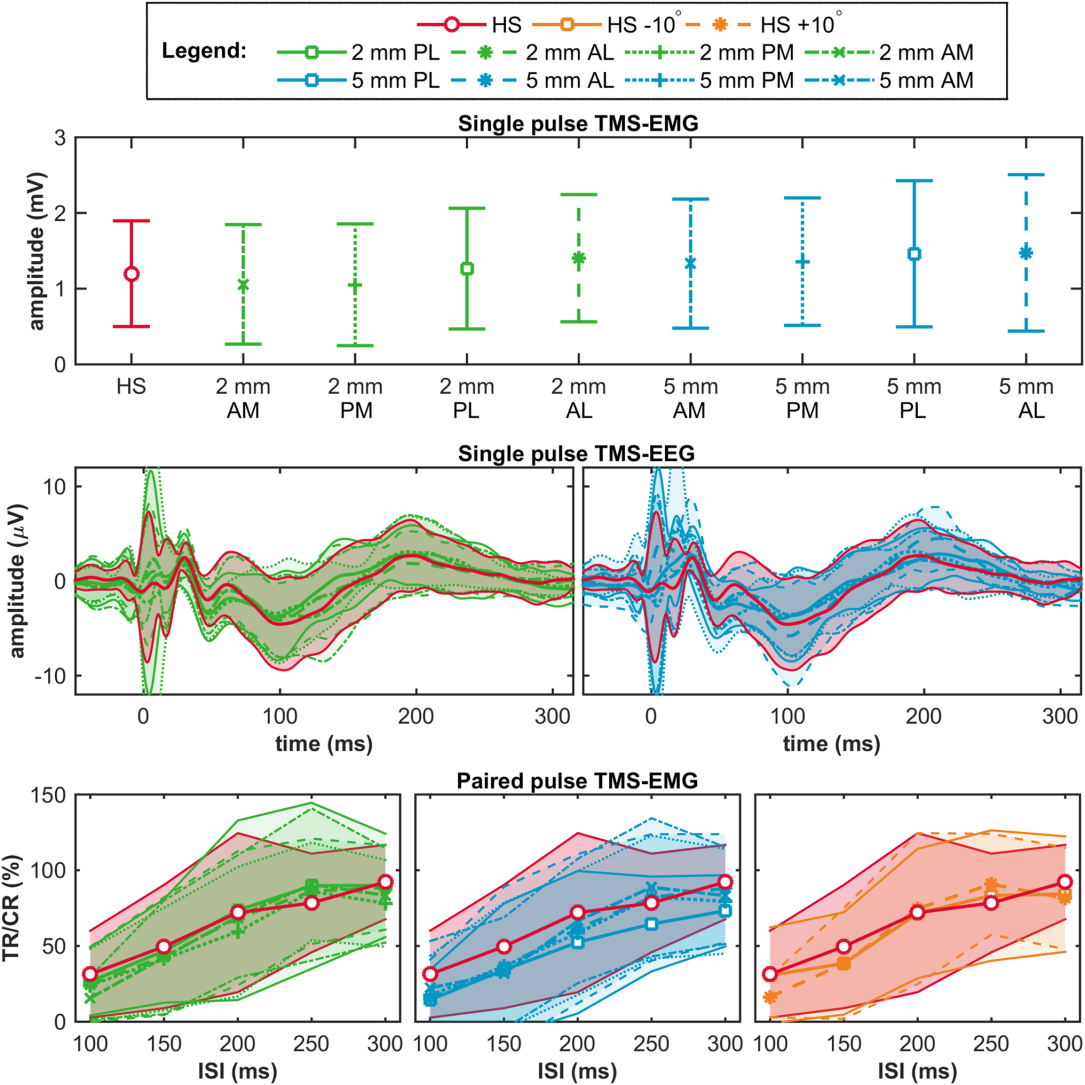
Outcomes at the group level. The top row shows per target the mean ± SD of the mean MEP amplitude per subject in the single pulse TMS-EMG study. The middle row shows per target the mean ± SD of the TEP at channel Cz per subject in the single pulse TMS-EEG study. The bottom row shows per target the mean ± SD of the LICI per subject in the paired pulse TMS-EMG study. In red, the outcomes measured at the hotspot (HS); in green, at a distance of 2 mm from the hotspot (HS); in blue, at a distance of 5 mm from the hotspot (HS); and in orange, for a 10° change in coil orientation. *AM* anterior-medial, *PM* posterior-medial, *PL* posterior-lateral, and *AL* anterior-lateral, *TR* test response, *CR* conditioning response, and *ISI* inter stimulus interval

At the group level, no significant differences were found for the mean MEP amplitudes measured at the nine stimulation targets (*F*(3.2,22.3) = 1.7, *p* = 0.20, $\eta _{p}^{2}$ = 0.19), nor for the amplitude variances (*F*(8,63) = 0.5, *p* = 0.86), see top row Fig. 3. For the MEP amplitudes (mean ± SD) measured at each target, see Table 2 in Supplementary material 1.

**Fig. 4:**
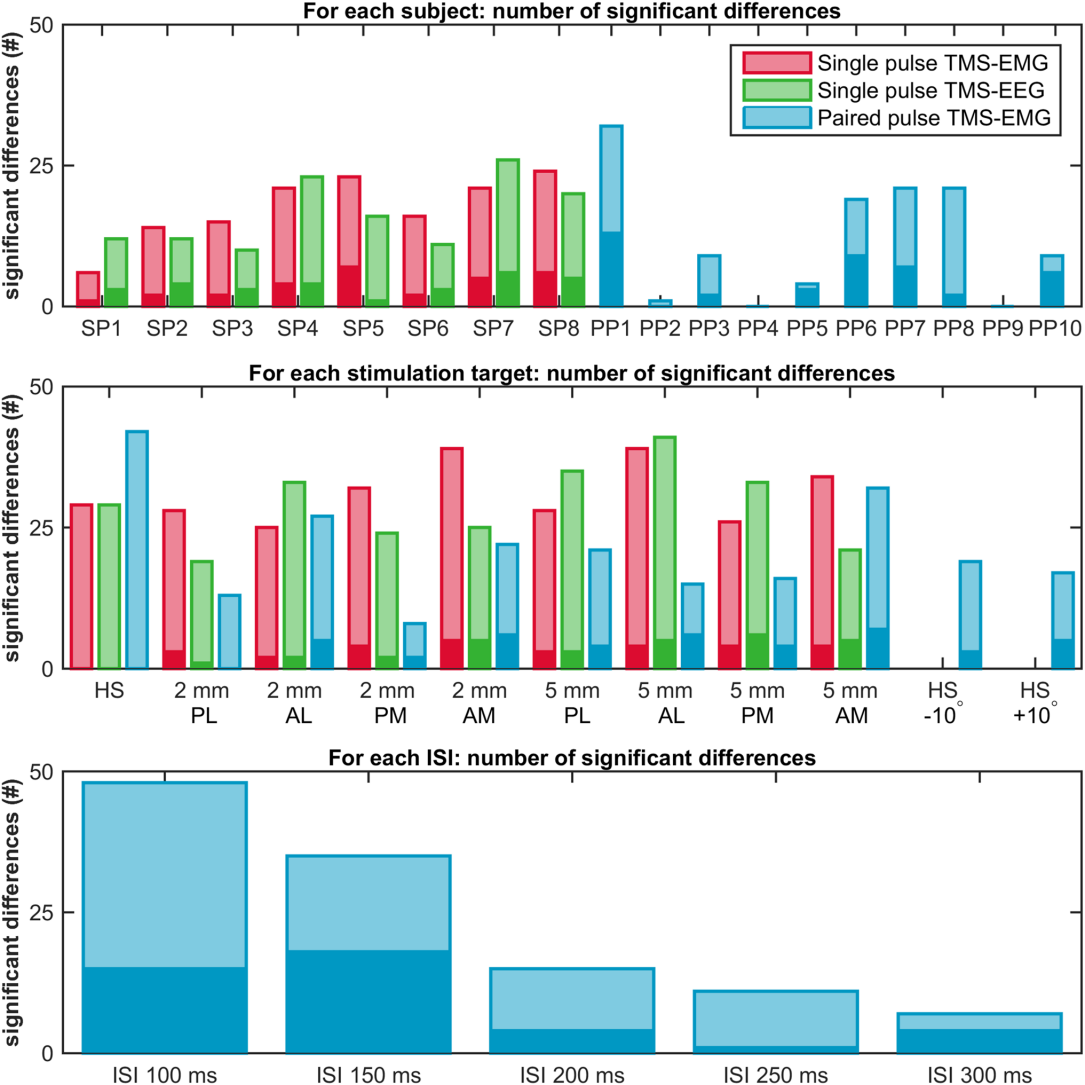
Outcomes at the subject level. Bar plots showing the number of significant differences found in each subject (top row), at each stimulation target (middle row), and for each ISI (bottom row). In red, results of the single pulse TMS-EMG study; in green, of the single pulse TMS-EEG study; and in blue, of the paired pulse TMS-EMG study. All significant differences combined are indicated by the light color bar, while differences for only the hotspot versus any other target are indicated by the dark color bar. *SP* single pulse, *PP* paired pulse, *HS* hotspot, *PL* posterior-lateral, *AL* anterior-lateral, *PM* posterior-medial, *AM* anterior-medial, and *ISI* inter stimulus interval

**Fig. 5:**
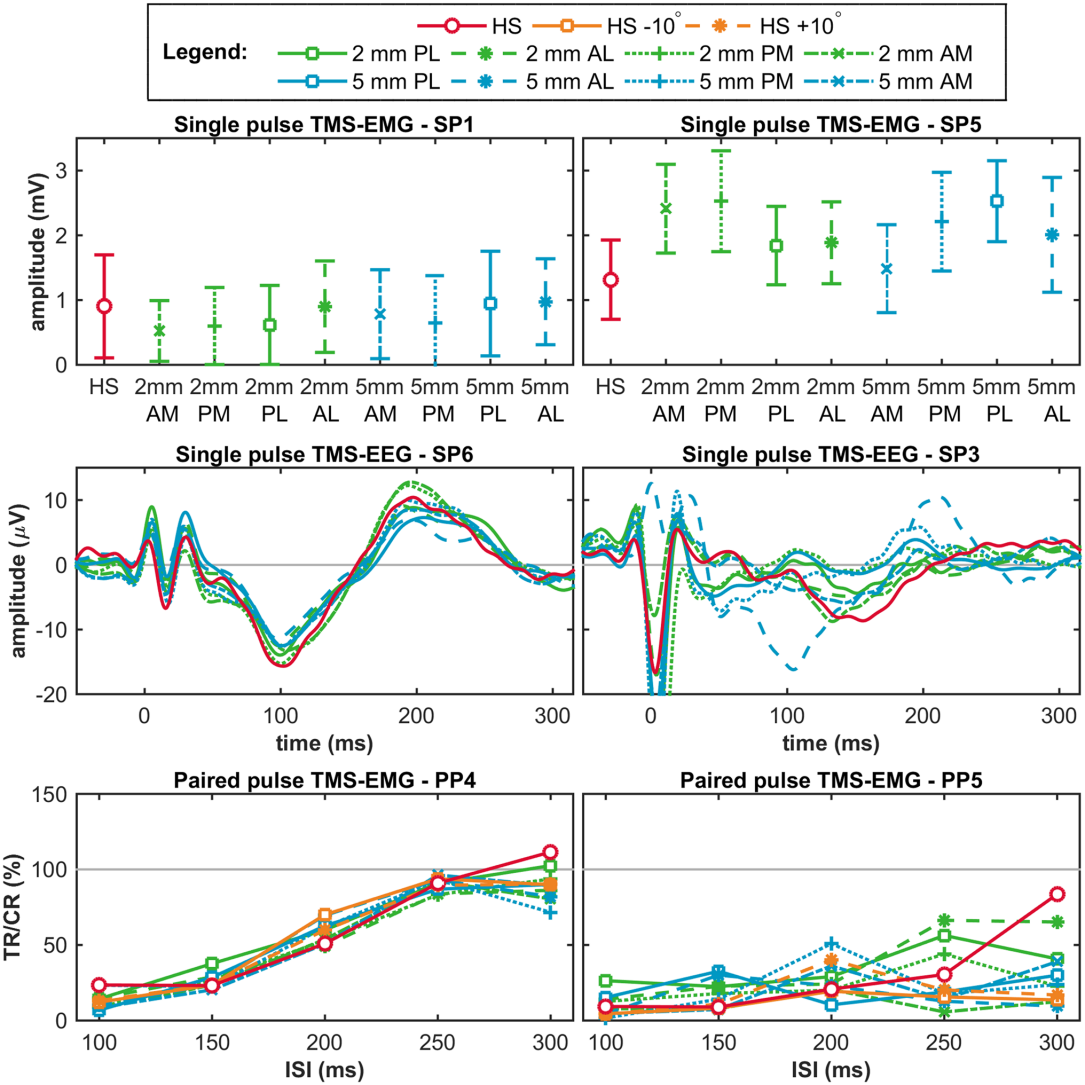
Examples of good (on the left) and poor (on the right) outcomes at the subject level. The top row shows two examples of the single pulse TMS-EMG study (mean ± SD MEP amplitude); the middle row of the single pulse TMS-EEG study (TEP at channel Cz); and the bottom row of the paired pulse TMS-EMG study (LICI curve). In red, outcomes measured at the hotspot (HS); in green, at a distance of 2 mm from the hotspot (HS); in blue, at a distance of 5 mm from the hotspot (HS); and in orange, for a 10 degree change in coil orientation. *SP* single pulse, *PP* paired pulse, *AM* anterior-medial, *PM* posterior-medial, *PL* posterior-lateral, and *AL* anterior-lateral, *TR* test response, *CR* conditioning response, and *ISI* inter stimulus interval

At the subject level, significant differences were found in all eight subjects (range 6–24 out of 36 possible comparisons), and for all nine targets (range 25–39 out of 64 possible comparisons), see light red bars in Fig. 4 and Table 2 in Supplementary material 1. Three out of eight subjects showed equality of variances (SP3: *F*(8,655) = 1.0, *p* = 0.42; SP5: *F*(8,661) = 1.3, *p* = 0.25; SP7: *F*(8,641) = 0.8, *p* = 0.61). Between subjects the absolute difference in mean MEP amplitude measured at two significant targets varied from 0.3 to 1.0 mV, while between targets the absolute difference for significant subjects in mean MEP amplitude measured at two significant targets varied from 0.5 to 0.7 mV, see red bars in Fig. 6 in Supplementary material 2. No clear differences were seen between the four locations at 2 mm distance and the four locations at 5 mm distance from the hotspot, nor between the medial and lateral targets, see dark red bars in Fig. 4. For two subjects the MEP amplitude at each of the nine stimulation targets is shown in the top row of Fig. 5.

### Single Pulse TMS-EEG Outcomes: TEP

At the group level, we found no significant effect of a change in coil location on the TEPs measured at the hotspot compared to targets surrounding the hotspot using cluster-based permutation analysis (no significant clusters), see middle row Fig. 3. Only one significant cluster (*p* < 0.001; parieto-occipital in the ipsilateral hemisphere between 40 and 90 ms) was found when comparing the TEPs measured at 2 mm PM and 5 mm PL.

At the subject level, significant clusters were found in all eight subjects (range 10–26 out of 36 possible comparisons), and for all nine targets (range 19–41 out of 64 possible comparisons), see light green bars in Fig. 4. No clear differences were seen between the four locations at 2 mm distance and the four locations at 5 mm distance from the hotspot, nor between the medial and lateral targets, see dark green bars in Fig. 4. Although significant clusters varied over subjects and targets, most clusters were found between 105 and 180 ms and involved electrodes in central and parietal brain areas. For a complete overview of the cluster characteristics, see Table 1. For two subjects the TEP at each of the nine stimulation targets is shown in the middle row of Fig. 5.

**Table 1:** Overview of the characteristics of the significant clusters found in the single pulse TMS-EEG study at the subject level

Cluster characteristic	Mean (±SD)
Electrodes involved (#)	20 ± 8
Length (ms)	73 ± 31
Start time (ms)	105 ± 72
Hemisphere involved	
Left (%)	26
Right (%)	10
Both (%)	65
Brain areas involved	
Frontal (%)	69
Central (%)	99
Parietal (%)	90
Temporal (%)	51
Occipital (%)	72

### Paired Pulse TMS-EMG Outcome: LICI

At the group level, no significant differences were found for LICI measured at any of the eleven stimulation targets (ISI 100 ms: *F*(3.8,26.4) = 2.0, *p* = 0.13, ${\upeta} _{\rm p}^{2}$ = 0.22; ISI 150 ms: *F*(2.5,17.7) = 1.2, *p* = 0.34, ${\upeta} _{\rm p}^{2}$ = 0.14; ISI 200 ms: *F*(2.7,19.3) = 0.8, *p* = 0.48, ${\upeta} _{\rm p}^{2}$ = 0.11; ISI 250 ms: *F*(3.1,21.9) = 1.2, *p* = 0.32, ${\upeta} _{\rm p}^{2}$ = 0.15; ISI 300 ms: *F*(3.5,24.5) = 0.9, *p* = 0.47, ${\upeta} _{\rm p}^{2}$ = 0.11), nor for the variance in LICI (ISI 100 ms: *p* = 0.35; ISI 150 ms: *p* = 0.98; ISI 200 ms: *p* = 0.99; ISI 250 ms: *p* = 0.78; ISI 300 ms: *p* = 0.96), see bottom row Fig. 3 and Tables 3, 4, 5, 6 and 7 in Supplementary material 1.

At the subject level, significant differences were found in eight out of ten subjects (range 1 to 32 out of 275 possible comparisons), for all 11 stimulation targets (range 8–42 out of 500 possible comparisons), and for all five ISIs (range 7–48 out of 550 possible comparisons), see light blue bars in Fig. 4 and Tables 3, 4, 5, 6 and 7 in Supplementary material 1. None of the ten subjects showed equality of variances for all five ISIs. Between subjects the absolute difference in LICI measured at two significant targets varied from 15 to 65%, while between targets the absolute difference for significant subjects in LICI measured at two significant targets varied from 26 to 48%, and between ISIs the absolute difference for significant subjects in LICI measured at two significant targets varied from 30 to 55%, see blue bars in Fig. 6 in Supplementary material 2. No clear differences were seen between the four locations at 2 mm distance and the four locations at 5 mm distance from the hotspot, nor between the medial and lateral targets, nor between an increase and decrease in coil angle, nor between ISIs, see dark blue bars in Fig. 4. For two subjects, the LICI curves measured at each of the eleven stimulation targets are shown in the bottom row of Fig. 5.

## Discussion

In this study, we investigated the accuracy of function-guided navigation for determining the hotspot by evaluating the effect of small changes in coil position on single and paired pulse excitability measures. For single pulse TMS, a change in coil location did not significantly affect the mean MEP amplitudes and TEPs at the group level. Only one significant cluster was found when comparing the TEPs measured at 2 mm PM and 5 mm PL. However, at the subject level both the MEP amplitudes and EEG responses were affected. Significant differences were found in all subjects and for all targets, while the degree of change (2 or 5 mm) and the direction of change (medial or lateral) made no difference. Similarly, for paired pulse TMS-EMG a change in coil location or orientation did not affect LICI at the group level, but at the subject level significant differences were found in eight out of ten subjects, for all targets, and for all ISIs.

### Accuracy of Hotspot Determination

In none of the subjects, the largest MEP amplitudes were evoked at the hotspot. When combining both studies, significant higher amplitudes were found at a different location in 15 out of 18 subjects. We determined the hotspot by function-guided navigation, so based on visual analysis of the MEP amplitude while stimulating the presumed hotspot area. Hereafter, robot-guided coil positioning was used to stimulate targets at 2 and 5 mm surrounding the hotspot, thereby ensuring accurate and stable positioning of the TMS coil at all of these targets.

To our knowledge the accuracy of function-guided navigation has not been studied before. A previous study by Komssi et al. (2002) stimulated four targets at 1 cm surrounding the hotspot, which was determined using function-guided navigation. However, they evaluated the TEP responses and did not report on the MEP amplitudes measured at the surrounding targets (Komssi et al. 2002). When comparing function-guided navigation to neuronavigation, the latter usually results in motor evoked potentials (MEPs) with significantly higher amplitudes (Gugino et al. 2001; Sparing et al. 2008; Julkunen et al. 2009; Jung et al. 2010). Neuronavigation uses brain imaging data to position the coil above the selected hotspot by considering inter-individual anatomical variability, instead of visual analysis of the MEP amplitude in the motor area. The fact that we were apparently not always able to determine the exact hotspot with function-guided navigation, may have resulted from the limited number of TMS pulses applied during the procedure and the normal variation in MEP amplitude, which is known to be large (Wassermann 2002; Goldsworthy et al. 2016).

### Effect of a Change in Coil Positioning

We showed that small changes in coil location or orientation can result in significantly different MEP amplitudes, LICI curves or TEPs at the subject level. However, no significant differences in mean values or variances were found at the group level. In general, the fact that no significant differences were found at the group level might be explained by our small sample size. However, others found significant differences for small groups (n = 6–8) (Komssi et al. 2002; Julkunen et al. 2009; Jung et al. 2010). Although previous studies reported significantly smaller MEP and TEP amplitudes at the group level for changes in coil position (Komssi et al. 2002; Sparing et al. 2008; Julkunen et al. 2009; Jung et al. 2010; Casarotto et al. 2010), their variations in coil location (> 1 cm) and orientation (> 45°) were much larger than in our study (2 or 5 mm and 10°, respectively). At the group level, function-guided navigation in combination with manual positioning and holding of the coil during stimulation seems to be sufficient accurate, if the changes in location and orientation remain respectively less than 10 mm and 20°. In clinical practice, this is feasible for experienced investigators. Julkunen et al. (2009) found that when no external coil fixation is used during stimulation, coil movement is on average < 10 mm from the mapped target among 20 consecutive TMS pulses (inter-pulse interval 4–6 s).

Most TMS studies report findings on a group level, either comparing patients to controls, or pre- to post-intervention TMS sessions. Our findings indicate that in such studies function-guided navigation can be combined with positioning and holding of the coil by hand or using a stand during stimulation, even though the accuracy and stability is lower than using frameless stereotaxic neuronavigation. The most important factor to take into account when deciding which type of navigation to use is the expected difference in excitability between conditions. For example, Appendix B shows a maximal increase or decrease in LICI of around 65% due to a change in coil positioning. However, differences between patients with epilepsy and healthy controls at ISI 250 ms have been reported to be three times as large: inhibition in controls (mean ± SD: 68.3 ± 37.0%), but facilitation in patients (generalized epilepsy: 273.0 ± 106.2%, and ipsilateral hemisphere focal epilepsy: 244.0 ± 76.4%) (Badawy et al. 2007, 2014, 2017; de Goede et al. 2016). Furthermore, in successfully treated patients facilitation may normalize to inhibition over time, while LICI remains increased in refractory patients (Badawy et al. 2013). Since the effects of changes in coil positioning on LICI are so much smaller than the expected LICI differences between the two test conditions, neuronavigation methods seem to be superfluous in epilepsy studies.

Our results also indicate that at the subject level it is preferred to use a coil positioning method with a high accuracy and stability, such as frameless stereotaxic neuronavigation. Again, the required accuracy in coil positioning depends on the disease and study design. For clinical applications decisions have to be made based on single subject data, comparing it to either reference values for diagnostic purposes, or to earlier measurements obtained in the same subject for follow-up or treatment evaluation purposes. Ultimately, TMS can only be used as a clinical tool if the differences in excitability between two conditions are larger than the intra-subject variability. In most subjects we found significant differences in measured variance, were the lowest variance was usually not measured at the hotspot determined by function-guided navigation. Although obtaining more stable MEPs using neuronavigation, the intra-subject and inter-subject variability remains high (Gugino et al. 2001; Julkunen et al. 2009; Jung et al. 2010).

The presence of significant differences varied over subjects and targets, but we could not find a pattern in the effects of a change in coil location or orientation. In some subjects, a change in coil positioning led to more significant differences than in others, indicating large inter-subject differences in the response to TMS. For the TMS-EMG paradigm this could be related to variations in individual sulcus anatomy (Balslev et al. 2007; Kallioniemi et al. 2015). For TMS-EEG, however, it is unclear whether sulcus anatomy would have such a large impact on the TEP, as it shows similar components when stimulating the motor or premotor cortex (Massimini 2005; Ferrarelli et al. 2008). Thus, the TEP seems to be a generic response that does not depend that much on the exact stimulation location. The fact that we did find significant differences in single subjects, but not on the group level might also be explained by the applied statistical analysis method. In TMS studies cluster-based permutation analysis is mostly used on the group level, and it may not be the most optimal method for single trial comparisons, although it is designed for single subject analysis (Maris and Oostenveld 2007). Related to this, the high sensitivity of the analysis method may explain the large variety in significant clusters that we found, both in size, length as well as location.

Although the presence of significant differences varied over targets, no clear differences were seen between the four locations at 2 mm distance and the four locations at 5 mm distance from the hotspot, nor between medial and lateral targets, nor between an increase and decrease in coil angle orientation. These findings suggest that the direction of change in location makes no difference, while Komssi et al. (2002) especially found attenuation of the TEP towards more medial stimulation sites. However, our changes in location were smaller and we only evaluated absence or presence of a significant difference.

### Limitations

This study is limited by the fact that our robot-guided navigation system only allowed adjustment of the coil location and orientation, but not the tilt. Thus, we could not evaluate the effect of a change in tilt on single and paired pulse excitability measures. Nevertheless, a recent study showed that excitability measures are mainly influenced by variations in coil location (36%), and not so much by tilt (5%) or orientation (< 1%) (Schmidt et al. 2015). However, in TMS-EEG studies varying the tilt might be of importance. When targets close to muscles are stimulated, associated muscle artifacts can be effectively reduced by rotating or tilting the coil wings away from the temporal muscle, while preserving the brain responses (Mutanen et al. 2013).

Even though the measurement protocol could have been optimized by randomizing the order of stimulation targets, our fixed sequence did probably not affect the outcomes. While at the subject level we found significant differences for all targets, clear differences between targets stimulated at the beginning or end of the study that may occur due to a gradual change in attention were not observed.

Furthermore, to limit the total measurement time, we applied ten paired pulses at each ISI. Although it is unknown how many pulses are needed for LICI estimation, a minimum of 20 and 25 pulses is needed for reliable estimation of short intracortical inhibition and intracortical facilitation (Chang et al. 2016), and at least 20 to 30 trials for single pulse TMS (Goldsworthy et al. 2016). The large number of 75 single pulses was needed for reliable estimation of the TEP. In contrast to Pellicciari et al. (2016), we found no cumulative effects for MEP amplitude over time, indicated by a coefficient of determination R^2^ always smaller than 0.5, that could have influenced our findings.

In conclusion, the accuracy of function-guided navigation for determining the hotspot is poor. In none of the subjects, the largest MEP amplitudes were evoked at the presumed hotspot. At the group level a change in coil location had no significant effect on the mean MEP amplitude, LICI and TEP or on the corresponding variances. In addition, a change in coil orientation did not significantly affect LICI using paired pulse TMS. At the subject level, significant differences in mean values and variances were found for both single and paired pulse TMS excitability measures, although absolute differences in MEP amplitude and LICI were relatively small. These findings indicate that a high accuracy in coil positioning is especially required to measure cortical excitability reliably over time in individual subjects using single or paired pulse TMS.

## Electronic Supplementary Material

Below is the link to the electronic supplementary material.

Supplementary material 1 (DOCX 32 KB)

Fig. 6 Outcomes at the subject level. Bar plots showng the absolute difference (mean ± SD) in mean MEP amplitude (single pulse TMS-EMG) or in LICI (paired pulse TMS-EMG) between two significant targets in each subject (top row), at each stimulation target (middle row), and for each ISI (bottom row). In red, results of the single pulse TMS-EMG study; and in blue, results of the paired pulse TMS-EMG study. SP = single pulse, PP = paired pulse, HS = hotspot, AM = anterior-medial, PM = posterior-medial, PL = posterior-lateral, AL = anterior-lateral, TR = test response, CR = conditioning response, and ISI = inter stimulus interval (TIFF 3430 KB)
