# Supplementary material for: Accurate Coil Positioning is Important for Single and Paired Pulse TMS on the Subject Level
Source: Brain Topogr. 2018 Jun 25;31(6):917–30. doi: 10.1007/s10548-018-0655-6 (PMC6182440; doi:10.1007/s10548-018-0655-6)
Supplement: Supplementary file 1 — Supplementary material 1 (DOCX 32 KB) [file 10548_2018_655_MOESM1_ESM.docx]

# Appendix A

***Table 2*** *Overview of the MEP amplitudes (mean ± SD) measured at each target at both the group and subject level in the single pulse TMS-EMG study. In addition, the (significant and non-significant) p-values are given for the performed ANOVA and Levene’s tests, as well as the effect sizes (*$\eta_{p}^{2}$*) for the ANOVA tests*

|  | **Group level** | **Subject level** | | | | | | | |
| --- | --- | --- | --- | --- | --- | --- | --- | --- | --- |
|  | **SP1-SP8** | **SP1** | **SP2** | **SP3** | **SP4** | **SP5** | **SP6** | **SP7** | **SP8** |
| **ANOVA** | *p* = 0.20  $\eta_{p}^{2}$ = 0.19 | *p* < 0.001  $\eta_{p}^{2}$ = 0.05 | *p* < 0.001  $\eta_{p}^{2}$ = 0.12 | *p* < 0.001  $\eta_{p}^{2}$ = 0.16 | *p* < 0.001  $\eta_{p}^{2}$ = 0.22 | *p* < 0.001  $\eta_{p}^{2}$ = 0.26 | *p* < 0.001  $\eta_{p}^{2}$ = 0.16 | *p* < 0.001  $\eta_{p}^{2}$ = 0.23 | *p* < 0.001  $\eta_{p}^{2}$ = 0.33 |
| **Levene’s** | *p* = 0.86 | *p* = 0.01 | *p* < 0.001 | *p* = 0.42 | *p* < 0.001 | *p* = 0.25 | *p* < 0.001 | *p* = 0.61 | *p* < 0.001 |
| **Target** | **mean ± SD (mV)** | **mean ± SD (mV)** | **mean ± SD (mV)** | **mean ± SD (mV)** | **mean ± SD (mV)** | **mean ± SD (mV)** | **mean ± SD (mV)** | **mean ± SD (mV)** | **mean ± SD (mV)** |
| hotspot | 1.2 ± 0.7 | 0.9 ± 0.8 | 0.9 ± 0.7 | 1.8 ± 0.8 | 0.3 ± 0.3 | 1.3 ± 0.6 | 0.5 ± 0.3 | 2.4 ± 0.8 | 1.5 ± 1.0 |
| 2 mm AM | 1.1 ± 0.8 | 0.5 ± 0.5 | 0.6 ± 0.5 | 1.0 ± 0.8 | 0.3 ± 0.4 | 2.4 ± 0.7 | 0.2 ± 0.2 | 1.8 ± 0.8 | 1.6 ± 0.9 |
| 2 mm PM | 1.1 ± 0.8 | 0.6 ± 0.6 | 0.4 ± 0.3 | 1.4 ± 0.6 | 0.1 ± 0.1 | 2.5 ± 0.8 | 0.5 ± 0.4 | 1.4 ± 0.7 | 1.5 ± 1.0 |
| 2 mm PL | 1.3 ± 0.8 | 0.6 ± 0.6 | 0.9 ± 0.7 | 1.8 ± 0.8 | 0.1 ± 0.2 | 1.8 ± 0.6 | 0.6 ± 0.4 | 2.3 ± 0.9 | 2.0 ± 0.9 |
| 2 mm AL | 1.4 ± 0.8 | 0.9 ± 0.7 | 0.9 ± 0.7 | 1.8 ± 0.7 | 0.3 ± 0.3 | 1.9 ± 0.6 | 0.6 ± 0.6 | 2.5 ± 0.8 | 2.4 ± 0.7 |
| 5 mm AM | 1.3 ± 0.9 | 0.8 ± 0.7 | 1.0 ± 0.6 | 2.3 ± 0.7 | 0.1 ± 0.1 | 1.5 ± 0.7 | 0.7 ± 0.6 | 1.7 ± 0.7 | 2.6 ± 0.6 |
| 5 mm PM | 1.4 ± 0.8 | 0.6 ± 0.7 | 0.9 ± 0.9 | 1.6 ± 0.7 | 0.1 ± 0.2 | 2.2 ± 0.8 | 0.9 ± 0.7 | 1.7 ± 0.7 | 2.6 ± 0.7 |
| 5 mm PL | 1.5 ± 1.0 | 0.9 ± 0.8 | 0.6 ± 0.5 | 1.5 ± 1.7 | 0.4 ± 0.4 | 2.5 ± 0.6 | 0.8 ± 0.6 | 1.9 ± 0.7 | 3.1 ± 0.7 |
| 5 mm AL | 1.5 ± 1.0 | 1.0 ± 0.7 | 0.4 ± 0.4 | 1.9 ± 0.8 | 0.6 ± 0.4 | 2.0 ± 0.9 | 0.4 ± 0.3 | 2.7 ± 0.9 | 2.9 ± 0.8 |

***Table 3*** *Overview of LICI for ISI 100 ms (mean ± SD) measured at each target at both the group and subject level in the paired pulse TMS-EMG study. In addition, the (significant and non-significant) p-values are given for the performed ANOVA and Bartlett’s tests, as well as the effect sizes (*$\eta_{p}^{2}$*) for the ANOVA tests*

|  | **Group level** | **Subject level** | | | | | | | | | |
| --- | --- | --- | --- | --- | --- | --- | --- | --- | --- | --- | --- |
|  | **PP1-PP10** | **PP1** | **PP2** | **PP3** | **PP4** | **PP5** | **PP6** | **PP7** | **PP8** | **PP9** | **PP10** |
| **ANOVA** | *p* = 0.13  $\eta_{p}^{2}$ = 0.22 | *p* < 0.001  $\eta_{p}^{2}$ = 0.35 | *p* = 0.003  $\eta_{p}^{2}$ = 0.24 | *p* = 0.41  $\eta_{p}^{2}$ = 0.10 | *p* = 0.17  $\eta_{p}^{2}$ = 0.13 | *p* = 0.05  $\eta_{p}^{2}$ = 0.18 | *p* < 0.001  $\eta_{p}^{2}$ = 0.32 | *p* < 0.001  $\eta_{p}^{2}$ = 0.37 | *p* < 0.001  $\eta_{p}^{2}$ = 0.37 | *p* = 0.03  $\eta_{p}^{2}$ = 0.20 | *p* < 0.001  $\eta_{p}^{2}$ = 0.39 |
| **Bartlett’s** | *p* = 0.35 | *p* < 0.001 | *p* = 0.04 | *p* < 0.001 | *p* < 0.001 | *p* < 0.001 | *p* < 0.001 | *p* < 0.001 | *p* < 0.001 | *p* = 0.02 | *p* = 0.01 |
| **Target** | **mean±SD (%)** | **mean±SD (%)** | **mean±SD (%)** | **mean±SD (%)** | **mean±SD (%)** | **mean±SD (%)** | **mean±SD (%)** | **mean±SD (%)** | **mean±SD (%)** | **mean±SD (%)** | **mean±SD (%)** |
| hotspot | 31.4 ± 28.7 | 39.4 ± 20.2 | 84.4 ± 23.1 | 2.8 ± 3.9 | 23.6 ± 14.5 | 9.3 ± 6.2 | 6.3 ± 8.8 | 52.9 ± 41.9 | 0.8 ± 0.6 | 29.0 ± 20.1 | 65.2 ± 36.4 |
| 2 mm PL | 26.5 ± 22.4 | 28.0 ± 21.8 | 75.2 ± 39.7 | 6.0 ± 5.8 | 14.3 ± 6.6 | 26.4 ± 38.1 | 9.8 ± 10.2 | 19.5 ± 13.3 | 0.6 ± 0.2 | 48.2 ± 27.3 | 37.3 ± 26.7 |
| 2 mm AL | 24.6 ± 24.8 | 8.6 ± 7.0 | 71.3 ± 22.7 | 4.0 ± 2.9 | 14.7 ± 9.7 | 14.0 ± 17.1 | 5.7 ± 5.9 | 51.3 ± 41.9 | 0.6 ± 0.3 | 53.2 ± 40.6 | 22.8 ± 11.7 |
| 2 mm PM | 24.0 ± 24.1 | 24.5 ± 18.5 | 77.9 ± 21.7 | 4.6 ± 2.9 | 8.9 ± 4.2 | 12.7 ± 10.1 | 2.1 ± 2.0 | 29.4 ± 21.4 | 0.5 ± 0.1 | 43.8 ± 28.3 | 35.4 ± 19.3 |
| 2 mm AM | 15.7 ± 14.1 | 14.2 ± 10.8 | 52.7 ± 39.2 | 7.0 ± 8.1 | 7.7 ± 4.4 | 7.8 ± 11.7 | 11.2 ± 8.2 | 15.4 ± 14.3 | 2.7 ± 2.5 | 18.6 ± 12.8 | 19.3 ± 12.5 |
| 5 mm PL | 14.5 ± 19.8 | 3.6 ± 4.4 | 65.5 ± 49.3 | 4.1 ± 4.2 | 6.8 ± 2.5 | 15.3 ± 22.7 | 6.4 ± 8.6 | 13.6 ± 20.3 | 0.4 ± 0.1 | 14.8 ± 13.9 |  |
| 5 mm AL | 15.4 ± 25.5 | 2.4 ± 1.4 | 80.0 ± 38.9 | 3.2 ± 2.2 | 10.3 ± 3.9 | 4.3 ± 2.8 | 6.8 ± 6.9 | 3.8 ± 4.0 | 0.7 ± 0.4 | 27.3 ± 26.6 |  |
| 5 mm PM | 17.5 ± 26.2 | 18.8 ± 16.6 | 84.0 ± 25.2 | 4.1 ± 6.1 | 23.4 ± 44.9 | 1.7 ± 1.5 | 3.0 ± 1.6 | 9.0 ± 10.2 | 0.4 ± 0.1 | 13.0 ± 14.1 |  |
| 5 mm AM | 22.3 ± 30.9 | 13.3 ± 17.5 | 99.8 ± 24.6 | 4.6 ± 4.7 | 8.5 ± 4.3 | 4.6 ± 3.0 | 27.9 ± 27.9 | 8.0 ± 9.8 | 1.7 ± 1.6 | 32.6 ± 29.2 |  |
| hotspot +10° | 30.6 ± 31.6 | 49.0 ± 42.6 | 90.2 ± 24.0 | 2.3 ± 1.3 | 12.1 ± 6.5 | 4.5 ± 3.7 | 7.1 ± 2.3 | 11.2 ± 7.9 |  | 32.0 ± 36.8 | 67.3 ± 25.6 |
| hotspot -10° | 16.4 ± 13.4 | 15.2 ± 21.8 | 34.6 ± 15.9 | 2.5 ± 2.1 | 13.2 ± 7.3 | 4.3 ± 4.0 | 1.3 ± 1.0 | 15.1 ± 15.0 |  | 38.8 ± 35.5 | 22.2 ± 29.5 |

***Table 4*** *Overview of LICI for ISI 150 ms (mean ± SD) measured at each target at both the group and subject level in the paired pulse TMS-EMG study. In addition, the (significant and non-significant) p-values are given for the performed ANOVA and Bartlett’s tests, as well as the effect sizes (*$\eta_{p}^{2}$*) for the ANOVA tests*

|  | **Group level** | **Subject level** | | | | | | | | | |
| --- | --- | --- | --- | --- | --- | --- | --- | --- | --- | --- | --- |
|  | **PP1-PP10** | **PP1** | **PP2** | **PP3** | **PP4** | **PP5** | **PP6** | **PP7** | **PP8** | **PP9** | **PP10** |
| **ANOVA** | *p* = 0.34  $\eta_{p}^{2}$ = 0.14 | *p* < 0.001  $\eta_{p}^{2}$ = 0.35 | *p* = 0.11  $\eta_{p}^{2}$ = 0.15 | *p* = 0.001  $\eta_{p}^{2}$ = 0.25 | *p* = 0.49  $\eta_{p}^{2}$ = 0.09 | *p* = 0.10  $\eta_{p}^{2}$ = 0.15 | *p* < 0.001  $\eta_{p}^{2}$ = 0.41 | *p* < 0.001  $\eta_{p}^{2}$ = 0.31 | *p* < 0.001  $\eta_{p}^{2}$ = 0.35 | *p* = 0.47  $\eta_{p}^{2}$ = 0.10 | *p* = 0.007  $\eta_{p}^{2}$ = 0.24 |
| **Bartlett’s** | *p* = 0.98 | *p* < 0.001 | *p* < 0.001 | *p* = 0.62 | *p* = 0.60 | *p* < 0.001 | *p* < 0.001 | *p* < 0.001 | *p* < 0.001 | *P* = 0.51 | *p* = 0.15 |
| **Target** | **mean±SD (%)** | **mean±SD (%)** | **mean±SD (%)** | **mean±SD (%)** | **mean±SD (%)** | **mean±SD (%)** | **mean±SD (%)** | **mean±SD (%)** | **mean±SD (%)** | **mean±SD (%)** | **mean±SD (%)** |
| hotspot | 49.6 ± 40.7 | 69.9 ± 39.0 | 123.4 ± 30.1 | 46.2 ± 24.5 | 23.2 ± 13.4 | 9.0 ± 7.6 | 34.7 ± 23.9 | 22.8 ± 17.0 | 1.8 ± 1.2 | 57.8 ± 41.3 | 107.8 ± 10.7 |
| 2 mm PL | 46.7 ± 34.1 | 44.6 ± 36.5 | 120.2 ± 35.3 | 47.0 ± 31.1 | 37.7 ± 16.8 | 22.5 ± 24.4 | 22.8 ± 13.3 | 27.4 ± 17.6 | 0.7 ± 0.3 | 67.3 ± 48.3 | 77.2 ± 27.0 |
| 2 mm AL | 43.2 ± 38.9 | 24.4 ± 19.3 | 137.0 ± 40.4 | 47.0 ± 20.6 | 28.9 ± 16.9 | 22.7 ± 29.4 | 10.9 ± 9.6 | 33.0 ± 22.5 | 1.0 ± 1.0 | 66.8 ± 49.9 | 60.8 ± 21.9 |
| 2 mm PM | 41.4 ± 33.8 | 38.2 ± 25.7 | 112.6 ± 21.7 | 61.6 ± 34.4 | 26.8 ± 22.8 | 17.9 ± 23.7 | 15.1 ± 14.0 | 14.7 ± 11.1 | 0.5 ± 0.1 | 64.7 ± 51.2 | 62.2 ± 16.8 |
| 2 mm AM | 42.4 ± 37.6 | 33.7 ± 45.4 | 113.1 ± 26.6 | 72.1 ± 24.1 | 23.6 ± 14.3 | 7.9 ± 7.0 | 9.9 ± 12.5 | 17.7 ± 16.4 | 1.2 ± 0.8 | 73.6 ± 50.0 | 71.8 ± 30.7 |
| 5 mm PL | 34.2 ± 43.9 | 6.0 ± 6.4 | 143.5 ± 100.7 | 19.7 ± 14.9 | 28.6 ± 10.4 | 32.7 ± 43.2 | 17.6 ± 13.5 | 7.8 ± 10.5 | 0.5 ± 0.2 | 51.3 ± 50.4 |  |
| 5 mm AL | 36.3 ± 52.8 | 7.4 ± 8.9 | 172.5 ± 89.6 | 34.5 ± 27.4 | 23.6 ± 11.8 | 30.1 ± 24.8 | 6.9 ± 13.2 | 11.7 ± 12.9 | 0.9 ± 0.9 | 38.8 ± 41.1 |  |
| 5 mm PM | 36.6 ± 42.0 | 19.2 ± 22.1 | 120.9 ± 20.5 | 42.5 ± 23.9 | 22.4 ± 16.6 | 14.1 ± 16.0 | 4.2 ± 5.3 | 13.3 ± 13.2 | 0.7 ± 0.8 | 91.8 ± 60.6 |  |
| 5 mm AM | 32.0 ± 36.8 | 11.2 ± 15.7 | 103.9 ± 19.5 | 68.7 ± 31.3 | 20.4 ± 12.5 | 7.3 ± 6.0 | 2.9 ± 0.7 | 7.1 ± 8.5 | 4.2 ± 4.2 | 62.3 ± 37.0 |  |
| hotspot +10° | 38.5 ± 33.8 | 47.1 ± 27.6 | 96.3 ± 10.3 | 39.2 ± 24.5 | 23.6 ± 13.7 | 8.2 ± 11.0 | 2.9 ± 3.0 | 4.3 ± 4.0 |  | 40.0 ± 22.6 | 84.6 ± 33.9 |
| hotspot -10° | 38.4 ± 36.5 | 15.6 ± 21.1 | 116.7 ± 32.1 | 48.1 ± 28.9 | 23.8 ± 16.3 | 10.9 ± 15.7 | 3.9 ± 3.8 | 8.5 ± 7.5 |  | 62.7 ± 53.3 | 55.4 ± 40.3 |

***Table 5*** *Overview of LICI for ISI 200 ms (mean ± SD) measured at each target at both the group and subject level in the paired pulse TMS-EMG study. In addition, the (significant and non-significant) p-values are given for the performed ANOVA and Bartlett’s tests, as well as the effect sizes (*$\eta_{p}^{2}$*) for the ANOVA tests*

|  | **Group level** | **Subject level** | | | | | | | | | |
| --- | --- | --- | --- | --- | --- | --- | --- | --- | --- | --- | --- |
|  | **PP1-PP10** | **PP1** | **PP2** | **PP3** | **PP4** | **PP5** | **PP6** | **PP7** | **PP8** | **PP9** | **PP10** |
| **ANOVA** | *p* = 0.48  $\eta_{p}^{2}$ = 0.11 | *p* < 0.001  $\eta_{p}^{2}$ = 0.39 | *p* = 0.99  $\eta_{p}^{2}$ = 0.02 | *p* = 0.01  $\eta_{p}^{2}$ = 0.20 | *p* = 0.48  $\eta_{p}^{2}$ = 0.09 | *p* = 0.49  $\eta_{p}^{2}$ = 0.09 | *p* = 0.007  $\eta_{p}^{2}$ = 0.25 | *p* = 0.33  $\eta_{p}^{2}$ = 0.10 | *p* = 0.05  $\eta_{p}^{2}$ = 0.17 | *p* = 0.85  $\eta_{p}^{2}$ = 0.06 | *p* = 0.27  $\eta_{p}^{2}$ = 0.11 |
| **Bartlett’s** | *p* = 0.99 | *p* = 0.002 | *p* = 0.01 | *p* = 0.32 | *p* = 0.27 | *p* = 0.03 | *p* < 0.001 | *p* = 0.66 | *p* = 0.49 | *p* = 0.24 | *p* = 0.19 |
| **Target** | **mean±SD (%)** | **mean±SD (%)** | **mean±SD (%)** | **mean±SD (%)** | **mean±SD (%)** | **mean±SD (%)** | **mean±SD (%)** | **mean±SD (%)** | **mean±SD (%)** | **mean±SD (%)** | **mean±SD (%)** |
| hotspot | 72.0 ± 52.5 | 64.4 ± 26.0 | 130.3 ± 36.3 | 52.5 ± 23.6 | 50.9 ± 20.2 | 20.8 ± 23.2 | 52.8 ± 36.5 | 30.9 ± 33.2 | 19.3 ± 10.0 | 173.5 ± 112.1 | 124.6 ± 39.7 |
| 2 mm PL | 73.6 ± 59.3 | 63.6 ± 28.3 | 127.0 ± 51.0 | 57.8 ± 20.8 | 59.9 ± 27.6 | 29.1 ± 47.8 | 42.1 ± 56.1 | 18.8 ± 22.7 | 15.6 ± 14.1 | 203.5 ± 111.8 | 118.2 ± 33.4 |
| 2 mm AL | 68.3 ± 44.2 | 63.2 ± 24.2 | 122.9 ± 37.9 | 74.0 ± 36.3 | 49.3 ± 23.5 | 24.2 ± 22.2 | 29.1 ± 27.7 | 50.9 ± 36.6 | 14.5 ± 11.6 | 143.2 ± 92.2 | 112.3 ± 26.2 |
| 2 mm PM | 59.4 ± 42.7 | 33.8 ± 28.5 | 116.6 ± 42.6 | 65.9 ± 26.6 | 52.9 ± 19.8 | 20.1 ± 24.7 | 23.9 ± 17.0 | 39.6 ± 26.6 | 10.7 ± 5.6 | 132.3 ± 112.5 | 98.6 ± 16.6 |
| 2 mm AM | 69.5 ± 40.4 | 32.7 ± 24.0 | 117.6 ± 26.3 | 92.7 ± 46.6 | 53.8 ± 24.1 | 20.6 ± 43.9 | 58.9 ± 23.8 | 48.5 ± 41.7 | 27.3 ± 12.1 | 115.8 ± 104.0 | 127.7 ± 31.8 |
| 5 mm PL | 52.5 ± 47.0 | 18.3 ± 9.7 | 121.2 ± 62.8 | 39.9 ± 28.7 | 62.7 ± 15.9 | 10.4 ± 14.0 | 25.1 ± 22.9 | 40.1 ± 26.9 | 15.5 ± 10.9 | 139.1 ± 164.9 |  |
| 5 mm AL | 61.5 ± 49.3 | 21.1 ± 16.6 | 123.4 ± 51.3 | 81.4 ± 32.7 | 69.6 ± 30.1 | 23.0 ± 32.6 | 17.8 ± 4.8 | 48.9 ± 29.6 | 17.3 ± 12.2 | 150.9 ± 149.1 |  |
| 5 mm PM | 58.5 ± 40.9 | 15.3 ± 10.3 | 131.3 ± 13.5 | 62.4 ± 39.9 | 61.8 ± 32.4 | 50.9 ± 45.3 | 24.6 ± 24.7 | 43.7 ± 29.3 | 20.7 ± 12.3 | 1116.2 ± 57.2 |  |
| 5 mm AM | 66.5 ± 41.1 | 17.8 ± 14.0 | 121.5 ± 34.5 | 88.6 ± 22.1 | 50.7 ± 12.1 | 36.2 ± 32.7 | 83.2 ± 64.4 | 47.4 ± 31.0 | 23.9 ± 9.9 | 129.5 ± 86.4 |  |
| hotspot +10° | 71.4 ± 42.7 | 36.1 ± 28.6 | 117.1 ± 40.7 | 69.4 ± 27.3 | 70.0 ± 21.6 | 19.4 ± 30.5 | 35.8 ± 33.5 | 44.3 ± 19.3 |  | 144.6 ± 132.3 | 106.3 ± 19.7 |
| hotspot -10° | 74.7 ± 49.8 | 47.5 ± 39.5 | 109.9 ± 27.7 | 57.1 ± 36.0 | 59.9 ± 26.1 | 40.1 ± 50.6 | 14.9 ± 12.6 | 54.8 ± 26.7 |  | 179.8 ± 88.7 | 108.2 ± 27.8 |

***Table 6*** *Overview of LICI for ISI 250 ms (mean ± SD) measured at each target at both the group and subject level in the paired pulse TMS-EMG study. In addition, the (significant and non-significant) p-values are given for the performed ANOVA and Bartlett’s tests, as well as the effect sizes (*$\eta_{p}^{2}$*) for the ANOVA tests*

|  | **Group level** | **Subject level** | | | | | | | | | |
| --- | --- | --- | --- | --- | --- | --- | --- | --- | --- | --- | --- |
|  | **PP1-PP10** | **PP1** | **PP2** | **PP3** | **PP4** | **PP5** | **PP6** | **PP7** | **PP8** | **PP9** | **PP10** |
| **ANOVA** | *p* = 0.32  $\eta_{p}^{2}$ = 0.15 | *p* < 0.001  $\eta_{p}^{2}$ = 0.27 | *p* = 0.27  $\eta_{p}^{2}$ = 0.12 | *p* = 0.04  $\eta_{p}^{2}$ = 0.17 | *p* = 1.00  $\eta_{p}^{2}$ = 0.02 | *p* = 0.10  $\eta_{p}^{2}$ = 0.15 | *p* = 0.26  $\eta_{p}^{2}$ = 0.13 | *p* = 0.02  $\eta_{p}^{2}$ = 0.18 | *p* < 0.001  $\eta_{p}^{2}$ = 0.34 | *p* = 0.35  $\eta_{p}^{2}$ = 0.11 | *p* = 0.65  $\eta_{p}^{2}$ = 0.06 |
| **Bartlett’s** | *p* = 0.78 | *p* = 0.01 | *p* < 0.001 | *p* = 0.77 | *p* = 0.70 | *p* < 0.001 | *p* = 0.97 | *p* = 0.001 | *p* = 0.04 | *p* = 0.16 | *p* = 0.36 |
| **Target** | **mean±SD (%)** | **mean±SD (%)** | **mean±SD (%)** | **mean±SD (%)** | **mean±SD (%)** | **mean±SD (%)** | **mean±SD (%)** | **mean±SD (%)** | **mean±SD (%)** | **mean±SD (%)** | **mean±SD (%)** |
| hotspot | 78.4 ± 32.6 | 51.2 ± 22.3 | 95.5 ± 36.2 | 71.2 ± 25.1 | 90.9 ± 23.8 | 30.5 ± 45.2 | 98.3 ± 35.2 | 34.1 ± 21.9 | 72.0 ± 32.4 | 127.8 ± 109.6 | 112.5 ± 37.6 |
| 2 mm PL | 89.8 ± 54.8 | 52.3 ± 39.4 | 92.8 ± 32.7 | 98.2 ± 27.5 | 90.7 ± 22.7 | 56.3 ± 54.2 | 103.4 ± 33.6 | 18.9 ± 12.2 | 53.2 ± 23.7 | 221.8 ± 121.9 | 110.0 ± 32.4 |
| 2 mm AL | 86.1 ± 34.8 | 54.8 ± 45.9 | 109.7 ± 49.3 | 88.1 ± 24.6 | 83.8 ± 38.6 | 66.3 ± 106.0 | 95.6 ± 34.9 | 25.2 ± 14.1 | 77.1 ± 19.9 | 154.7 ± 95.4 | 105.6 ± 22.5 |
| 2 mm PM | 86.0 ± 32.2 | 71.3 ± 35.5 | 109.4 ± 34.0 | 89.3 ± 23.5 | 83.3 ± 26.8 | 44.1 ± 72.2 | 126.1 ± 37.5 | 38.7 ± 21.8 | 59.9 ± 17.7 | 123.8 ± 99.6 | 114.4 ± 33.2 |
| 2 mm AM | 90.6 ± 50.2 | 51.6 ± 49.4 | 93.6 ± 15.3 | 109.4 ± 27.5 | 92.2 ± 33.5 | 5.7 ± 5.0 | 114.2 ± 29.1 | 42.7 ± 30.9 | 78.5 ± 21.6 | 183.9 ± 93.7 | 134.3 ± 41.6 |
| 5 mm PL | 64.5 ± 31.3 | 25.2 ± 14.4 | 70.3 ± 68.9 | 84.4 ± 38.3 | 86.9 ± 25.8 | 18.9 ± 30.7 | 90.2 ± 35.7 | 49.1 ± 34.9 | 45.7 ± 17.4 | 109.6 ± 71.0 |  |
| 5 mm AL | 82.0 ± 41.7 | 43.9 ± 29.8 | 144.2 ± 113.0 | 93.2 ± 17.8 | 93.0 ± 19.4 | 12.7 ± 17.2 | 105.9 ± 24.3 | 39.0 ± 22.4 | 91.7 ± 39.7 | 114.5 ± 35.8 |  |
| 5 mm PM | 82.3 ± 40.6 | 27.4 ± 18.7 | 110.1 ± 32.9 | 90.0 ± 26.8 | 93.1 ± 33.3 | 17.7 ± 15.5 | 118.1 ± 29.4 | 48.6 ± 44.7 | 109.4 ± 41.6 | 126.0 ± 103.9 |  |
| 5 mm AM | 88.7 ± 45.6 | 37.2 ± 22.3 | 101.4 ± 19.4 | 113.6 ± 21.5 | 96.3 ± 28.5 | 14.1 ± 14.3 | 137.6 ± 50.9 | 57.1 ± 33.1 | 87.8 ± 23.5 | 153.6 ± 127.5 |  |
| hotspot +10° | 83.3 ± 43.0 | 35.3 ± 28.2 | 117.0 ± 25.5 | 99.1 ± 26.7 | 93.8 ± 26.7 | 15.6 ± 20.3 | 123.0 ± 41.5 | 32.3 ± 15.1 |  | 119.4 ± 72.4 | 114.1 ± 28.2 |
| hotspot -10° | 90.8 ± 33.2 | 92.7 ± 29.0 | 98.9 ± 28.0 | 104.2 ± 29.5 | 89.5 ± 34.9 | 20.3 ± 25.5 | 99.6 ± 33.1 | 63.5 ± 38.1 |  | 141.0 ± 108.6 | 107.4 ± 49.5 |

***Table 7*** *Overview of LICI for ISI 300 ms (mean ± SD) measured at each target at both the group and subject level in the paired pulse TMS-EMG study. In addition, the (significant and non-significant) p-values are given for the performed ANOVA and Bartlett’s tests, as well as the effect sizes (*$\eta_{p}^{2}$*) for the ANOVA tests*

|  | **Group level** | **Subject level** | | | | | | | | | |
| --- | --- | --- | --- | --- | --- | --- | --- | --- | --- | --- | --- |
|  | **PP1-PP10** | **PP1** | **PP2** | **PP3** | **PP4** | **PP5** | **PP6** | **PP7** | **PP8** | **PP9** | **PP10** |
| **ANOVA** | *p* = 0.47  $\eta_{p}^{2}$ = 0.11 | *p* = 0.96  $\eta_{p}^{2}$ = 0.04 | *p* = 0.97  $\eta_{p}^{2}$ = 0.03 | *p* < 0.001  $\eta_{p}^{2}$ = 0.28 | *p* = 0.30  $\eta_{p}^{2}$ = 0.11 | *p* = 0.001  $\eta_{p}^{2}$ = 0.26 | *p* = 0.16  $\eta_{p}^{2}$ = 0.15 | *p* = 0.19  $\eta_{p}^{2}$ = 0.12 | *p* = 0.11  $\eta_{p}^{2}$ = 0.14 | *p* = 0.51  $\eta_{p}^{2}$ = 0.09 | *p* = 0.88  $\eta_{p}^{2}$ = 0.04 |
| **Bartlett’s** | *p* = 0.96 | *p* = 0.13 | *p* = 0.15 | *p* = 0.78 | *p* = 0.51 | *p* < 0.001 | *p* = 0.98 | *p* = 0.003 | *p* = 0.67 | *p* = 0.17 | *p* = 0.82 |
| **Target** | **mean±SD (%)** | **mean±SD (%)** | **mean±SD (%)** | **mean±SD (%)** | **mean±SD (%)** | **mean±SD (%)** | **mean±SD (%)** | **mean±SD (%)** | **mean±SD (%)** | **mean±SD (%)** | **mean±SD (%)** |
| hotspot | 92.2 ± 24.5 | 98.1 ± 49.0 | 105.1 ± 48.4 | 73.8 ± 20.7 | 111.5 ± 37.7 | 83.6 ± 75.1 | 106.1 ± 42.9 | 31.2 ± 23.0 | 110.9 ± 34.9 | 97.1 ± 88.9 | 104.5 ± 37.6 |
| 2 mm PL | 90.0 ± 34.1 | 100.8 ± 68.8 | 97.5 ± 39.0 | 71.9 ± 25.4 | 102.5 ± 34.6 | 40.8 ± 45.1 | 106.4 ± 32.9 | 30.7 ± 15.5 | 97.0 ± 30.5 | 148.0 ± 108.6 | 104.2 ± 38.4 |
| 2 mm AL | 88.6 ± 27.8 | 107.0 ± 42.0 | 107.7 ± 32.7 | 63.2 ± 22.5 | 86.2 ± 30.0 | 65.2 ± 71.9 | 115.1 ± 52.7 | 28.9 ± 16.0 | 106.1 ± 20.4 | 108.0 ± 83.2 | 98.6 ± 29.4 |
| 2 mm PM | 78.2 ± 28.6 | 73.9 ± 39.5 | 103.6 ± 30.3 | 77.3 ± 26.8 | 93.7 ± 23.5 | 22.8 ± 20.7 | 115.3 ± 33.5 | 41.7 ± 18.2 | 70.6 ± 19.2 | 49.3 ± 80.3 | 103.6 ± 240.1 |
| 2 mm AM | 83.4 ± 31.1 | 104.8 ± 90.1 | 102.1 ± 40.9 | 110.8 ± 22.1 | 80.5 ± 28.0 | 12.5 ± 19.7 | 99.7 ± 40.6 | 44.1 ± 32.2 | 98.0 ± 24.4 | 88.8 ± 90.8 | 92.8 ± 39.1 |
| 5 mm PL | 73.2 ± 23.6 | 92.4 ± 84.2 | 94.2 ± 65.7 | 64.7 ± 28.8 | 90.0 ± 20.7 | 30.0 ± 36.4 | 75.6 ± 47.9 | 52.2 ± 33.8 | 100.8 ± 31.1 | 59.2 ± 61.8 |  |
| 5 mm AL | 88.1 ± 35.7 | 108.9 ± 81.9 | 116.5 ± 43.1 | 94.1 ± 18.2 | 82.0 ± 22.0 | 9.5 ± 15.0 | 125.7 ± 37.1 | 56.2 ± 42.2 | 103.8 ± 26.5 | 95.7 ± 71.4 |  |
| 5 mm PM | 79.6 ± 34.6 | 91.4 ± 80.3 | 117.5 ± 26.4 | 83.8 ± 31.3 | 71.6 ± 24.9 | 23.8 ± 18.1 | 124.9 ± 41.6 | 31.9 ± 13.4 | 99.8 ± 34.0 | 71.4 ± 44.3 |  |
| 5 mm AM | 83.3 ± 32.0 | 66.3 ± 54.8 | 107.4 ± 23.7 | 96.4 ± 22.9 | 89.2 ± 32.8 | 39.0 ± 30.7 | 140.7 ± 40.6 | 41.5 ± 26.5 | 90.7 ± 27.6 | 78.1 ± 75.2 |  |
| hotspot +10° | 84.2 ± 38.1 | 97.1 ± 93.9 | 114.3 ± 45.9 | 90.7 ± 16.9 | 89.9 ± 34.7 | 13.6 ± 16.6 | 127.1 ± 39.5 | 30.1 ± 13.4 |  | 84.1 ± 44.1 | 111.3 ± 29.3 |
| hotspot -10° | 81.4 ± 33.5 | 86.8 ± 59.3 | 112.1 ± 34.4 | 81.8 ± 19.6 | 90.4 ± 42.3 | 16.9 ± 15.2 | 91.6 ± 38.9 | 39.1 ± 21.8 |  | 123.9 ± 112.7 | 90.0 ± 46.8 |
