# Supplementary figures and images for: Accurate Coil Positioning is Important for Single and Paired Pulse TMS on the Subject Level
Source: Brain Topogr. 2018 Jun 25;31(6):917–30. doi: 10.1007/s10548-018-0655-6 (PMC6182440; doi:10.1007/s10548-018-0655-6)

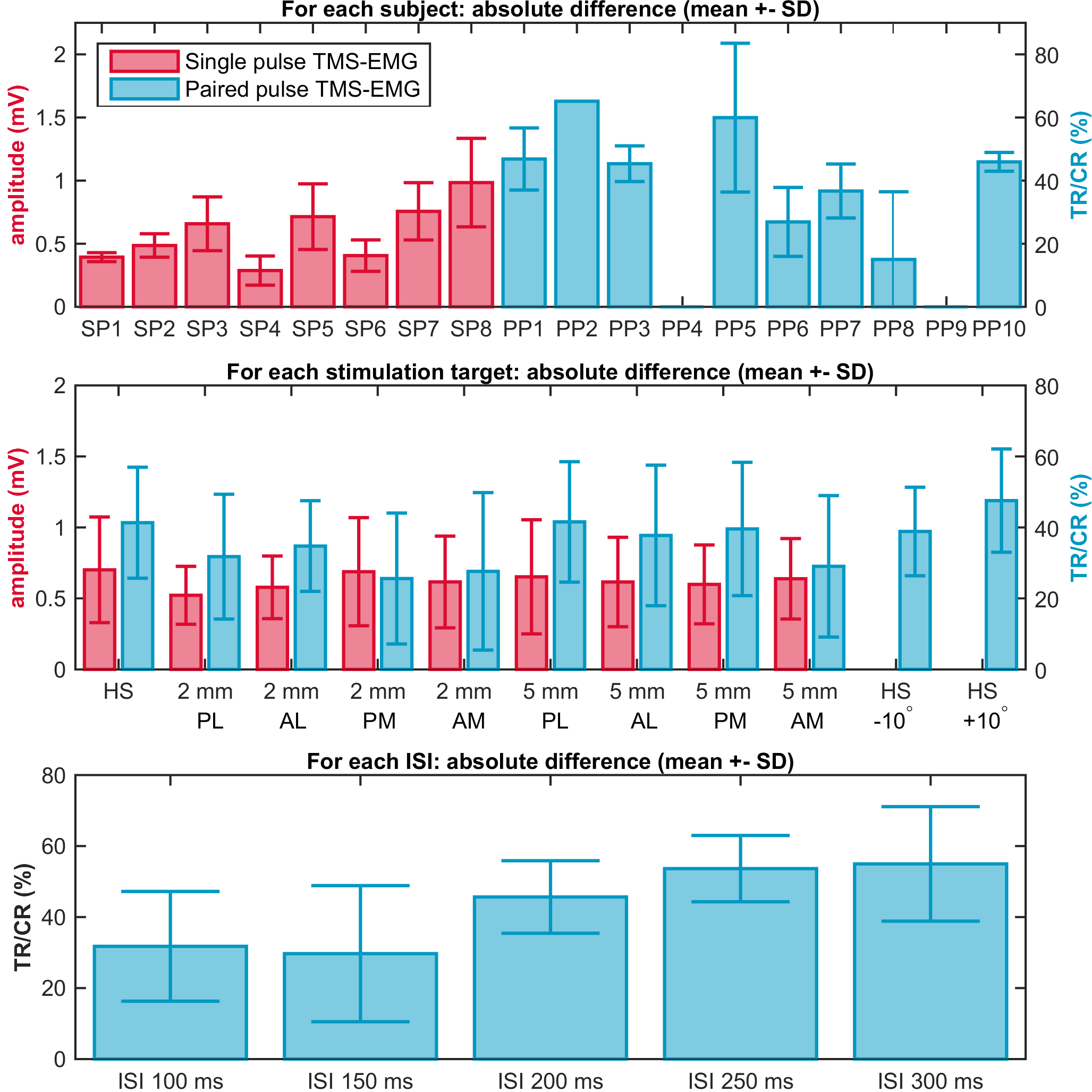

Supplement: Supplementary file 2 — Outcomes at the subject level. Bar plots showng the absolute difference (mean ± SD) in mean MEP amplitude (single pulse TMS-EMG) or in LICI (paired pulse TMS-EMG) between two significant targets in each subject (top row), at each stimulation target (middle row), and for each ISI (bottom row). In red, results of the single pulse TMS-EMG study; and in blue, results of the paired pulse TMS-EMG study. SP = single pulse, PP = paired pulse, HS = hotspot, AM = anterior-medial, PM = posterior-medial, PL = posterior-lateral, AL = anterior-lateral, TR = test response, CR = conditioning response, and ISI = inter stimulus interval (TIFF 3430 KB) [file 10548_2018_655_MOESM2_ESM.tiff]
